# Neural oscillations in the primate caudate nucleus correlate with different preparatory states for temporal production

**DOI:** 10.1038/s42003-019-0345-2

**Published:** 2019-03-14

**Authors:** Tomoki W. Suzuki, Masaki Tanaka

**Affiliations:** Department of Physiology, Hokkaido University School of Medicine, Sapporo, 060-8638 Japan

## Abstract

When measuring time, neuronal activity in the cortico-basal ganglia pathways has been shown to be temporally scaled according to the interval, suggesting that signal transmission within the pathways is flexibly controlled. Here we show that, in the caudate nuclei of monkeys performing a time production task with three different intervals, the magnitude of visually-evoked potentials at the beginning of an interval differed depending on the conditions. Prior to this response, the power of low frequency components (6–20 Hz) significantly changed, showing inverse correlation with the visual response gain. Although these components later exhibited time-dependent modification during self-timed period, the changes in spectral power for interval conditions qualitatively and quantitatively differed from those associated with the reward amount. These results suggest that alteration of network state in the cortico-basal ganglia pathways indexed by the low frequency oscillations may be crucial for the regulation of signal transmission and subsequent timing behavior.

## Abstract (web summary)

Tomoki Suzuki and Masaki Tanaka measured local field potentials in the caudate nucleus of monkeys performing a time production task and showed that the length of the time interval modified the magnitude of visually-evoked potentials and the spectral power at low frequencies. These changes suggest that neural oscillations within the cortico-basal ganglia pathways regulate timing behavior.

## Introduction

The basal ganglia play a central role in temporal information processing^1,2^. For example, individuals with Parkinson’s disease (PD) show impairments in a variety of timing tasks^1,3^, and functional imaging in healthy subjects detects increased activity in the basal ganglia during timing^4^. In experimental animals, neurons in the striatum exhibit a monotonic increase in firing rates during the monitoring of elapsed time^5–7^, and pharmacological manipulation of these signals alters the timing of self-initiated movements^8^. However, such ramping activity during motor preparation is ubiquitous in the cortico-basal ganglia-thalamic pathways^9–20^, and inactivation of respective recording sites also results in an alteration of timing behavior^21,22^. These previous observations indicate that, in addition to local computations, information processing through the global network must be crucial for the development and maintenance of preparatory activity^23^. In support of this, recent studies have shown that the time courses of neuronal activity both in the striatum and the cerebral cortex during timing tasks are temporally scaled in proportion to the length of the measured interval^24–26^, suggesting a close functional linkage between the recording sites. However, how the cortico-basal ganglia circuitry generates these signals remains largely unknown. To elucidate the underlying mechanism, the assessment of circuit-level computations appears to be necessary.

One way to explore the network state is to analyze the local field potentials (LFPs)^27^. Previous studies have shown that LFPs recorded from the cortico-basal ganglia pathways during dopamine depletion often exhibit pathological oscillation at low frequencies (approximately 8–30 Hz in PD patients^28^, 8–15 Hz in monkeys^29,30^, 4–30 Hz in rodents^30,31^), which has been suggested to be associated with an altered signal transmission^29,32,33^. Sporadic low-frequency oscillations in the cortico-basal ganglia pathways have also been reported in normal animals^34–36^. For example, one study demonstrated that the power of 10–25 Hz components in the striatum was dynamically modulated in monkeys performing a simple saccade task^37^. Such LFP modulations may reflect dynamic alteration of information flow through the cortico-basal ganglia pathways depending on the behavioral context^28,37–39^.

In the present study, we explored the context-dependent modulation of striatal LFP in monkeys performing a time production task, with the aim to understand the mechanism for the regulation of the temporal profile of timing-related neuronal activity. We found that the magnitudes of visually evoked potentials were different depending on the length of the interval, and that the spectral power at low frequencies also showed interval-dependent modification, even before monitoring elapsed time. These changes likely reflect a network alteration within the cortico-basal ganglia pathways that may causally regulate the timing of self-initiated movements.

## Results

**Fig. 1:**
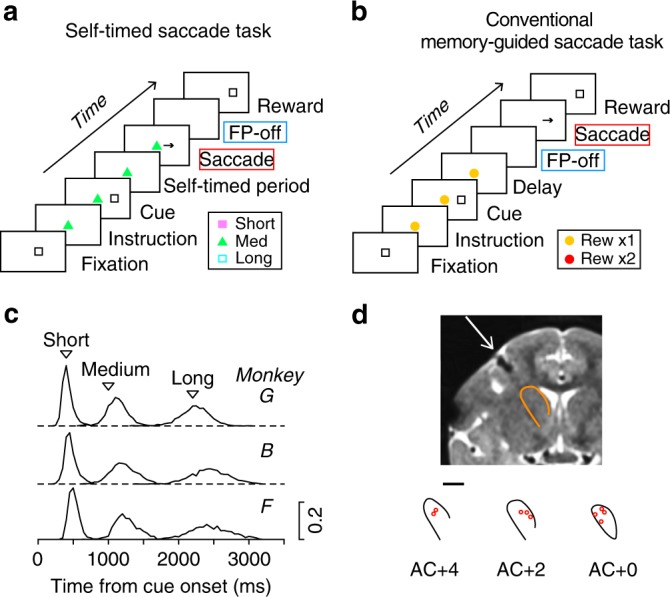
Behavioral paradigms and the locations of recording sites. **a** In the self-timed saccade task, monkeys were trained to generate a self-initiated saccade to the location of a briefly presented visual cue (100 ms) after the mandatory delay interval (400, 1000, or 2200 ms). **b** In the conventional memory-guided saccade (MS) task, animals made a saccade in response to the fixation point (FP) offset that occurred 800–2500 ms after the visual cue. Each task condition was indicated by specific color and shape of the FP, and the stimulus set for monkey F is shown here. A different set of the FP color and shape was used for the other two monkeys (see Methods). The visual cue appeared 800–1700 ms following the onset of this instruction. **c** Distributions of self-timed saccade latency during LFP recording in three monkeys (*n* = 3225, 4373, and 4588 trials for monkeys G, B, and F, respectively). Inverted triangles indicate the minimal mandatory intervals to obtain reward. **d** Coronal MR image (AC + 2) and recording sites (red circles) in monkey F. Scale bar represents 5 mm. Recording sites were reconstructed from histological sections in monkey G (Supplementary Figure 1). AC, anterior commissure

Three monkeys performed two types of memory-guided saccade (MS) tasks. In the self-timed saccade task (Fig. 1a)^22^, the animals obtained a reward when they generated a saccade after the predetermined delay interval following a visual cue. Different interval conditions were associated with different fixation point (FP) colors. As shown in the frequency distributions in Fig. 1c, monkeys successfully adjusted saccade timing depending on the instruction in each trial. In the conventional MS task (Fig. 1b)^40^, the animals were required to generate an immediate saccade in response to the FP offset, and therefore saccade timing was explicitly instructed. During these tasks, we recorded LFPs from the anterior part of the caudate nucleus (from 1 mm posterior to 4.5 mm anterior to the anterior commissure; Fig. 1d and Supplementary Figure 1), where we previously found preparatory spiking activity that started just after the cue onset and peaked around the time of saccades^7,8^.

### Context-dependent modification of visually evoked potentials

**Fig. 2:**
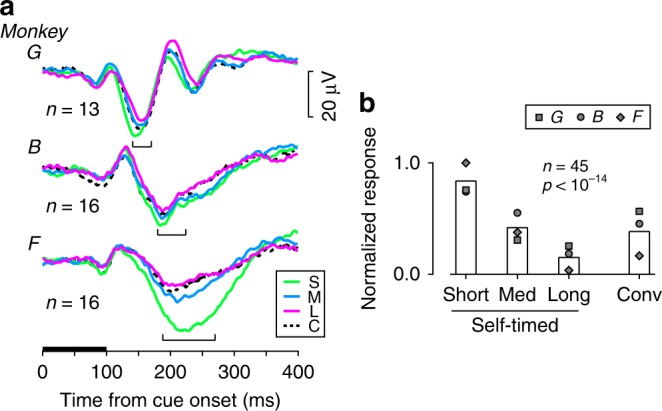
Contextual modulation of visually evoked potentials. **a** Time courses of striatal LFPs aligned with the cue onset in the contralateral visual field. Colored traces indicate the self-timed trials with different interval conditions. Black dashed traces indicate the conventional MS trials. The horizontal black bar denotes the timing of cue presentation. Brackets indicate the ranges of maximal response timing. **b** Comparison of the magnitude of visually evoked response. Each bar summarizes normalized response obtained from 45 sites. Different symbols plot the means of different monkeys. A one-way repeated measures ANOVA revealed a statistically significant difference across the interval conditions (*F*_2,88_ = 50.4, *p* < 10^−14^). Conv, conventional memory-guided saccade (MS) task

Figure 2a illustrates the average LFP traces for multiple experiments aligned with the cue appearance in the contralateral visual field. For all three monkeys, early small responses were followed by a large negative component that peaked at 140–169 ms (monkey G), 179–223 ms (B), and 187–269 ms (F) following the cue onset (brackets). While the visual cue was identical across conditions, this negative component tended to be larger for the short interval condition (green traces) and smaller for the long interval condition (magenta). When we measured the magnitude of the peak response for each site, the normalized values were statistically different across the three interval conditions in the self-timed trials (Fig. 2b; one-way repeated measures ANOVA, *F*_2,88_ = 50.4, *p* < 10^−14^). Post hoc paired *t*-tests showed that the values were statistically different between each pair of conditions (*t*_44_ = 4.09–11.14, *p* < 10^−3^).

### Spectral assessment of striatal LFPs

**Fig. 3:**
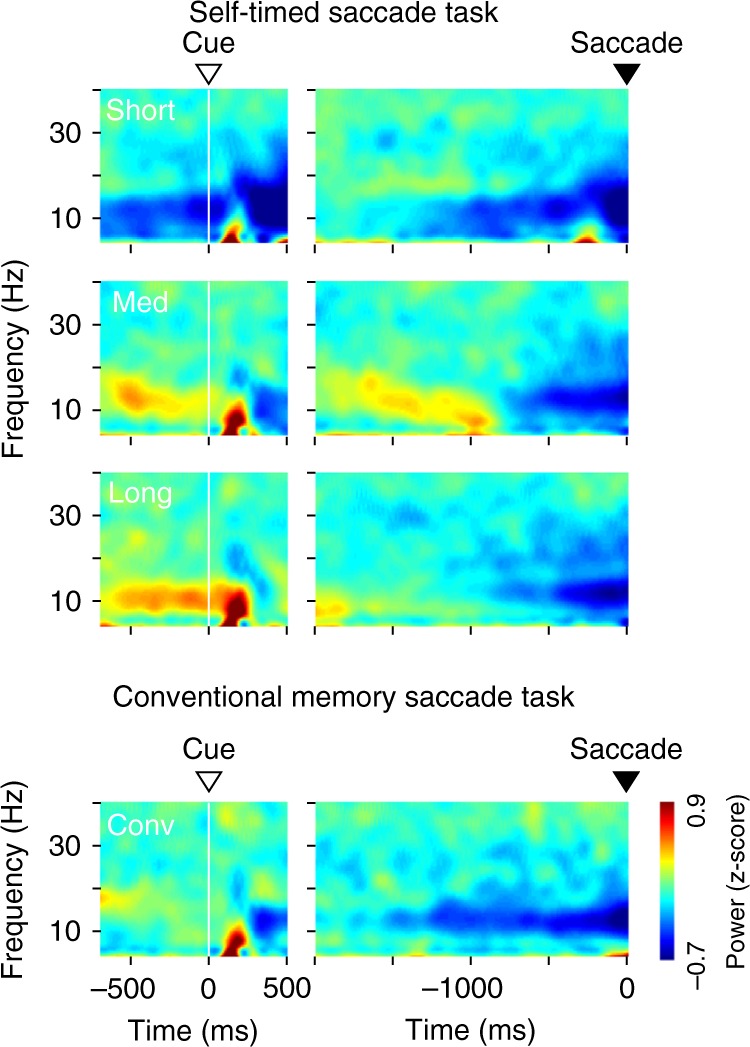
Color-coded power spectra of LFP in monkey G (*n* = 13 sites) for the self-timed and the conventional MS tasks. Zero in the abscissa indicates either the cue onset (left panels) or saccade initiation (right). Data for the other two monkeys are shown in Supplementary Figure 2

**Fig. 4:**
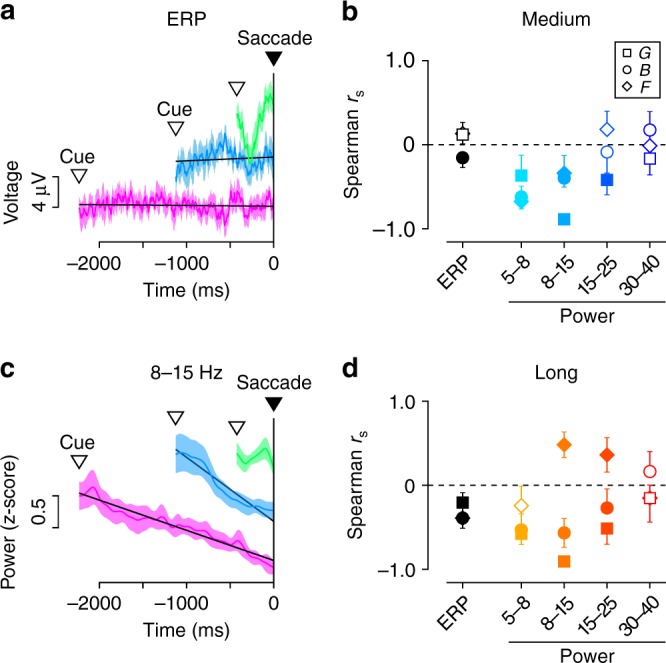
Temporally variable LFP components during the self-timed period in the self-timed task. **a** Time courses of event-related potentials (ERP) during saccade preparation in monkey G (*n* = 13 sites). Green, blue, and magenta traces indicate the means (±95% CIs) for the short, medium, and long interval conditions, respectively. Inverted open triangles indicate the means of cue onset time relative to saccades. The traces are shifted vertically for presentation purpose only. Black lines indicate linear regressions for the average traces in the medium and long interval conditions. **b** Spearman’s rank correlation coefficients (*r*_s_) computed between time (10 bins) and ERP or the power of different LFP components in the medium interval (1000 ms) condition. Different shapes of symbols represent different animals. Filled symbols indicate the data showing a significant difference from zero (one-sample *t*-test, *n* = 13 or 16, *p* < 0.05). Error bars indicate 95% CIs. **c** Similar configuration as in **a**, but for the time courses of LFP power at 8–15 Hz. **d** Rank correlation coefficients computed for the long interval (2200 ms) condition

Context-dependent alteration of visually evoked response suggests that the state of the cortico-striatal network might differ depending on the instruction. To explore this, we examined the spectral modulation of LFPs. Figure 3 displays the time courses of spectral power aligned with either the cue onset (left panels) or saccade initiation (right) in monkey G (*n* = 13 sites; Supplementary Figure 2 for the other animals). We found that the power of low-frequency components (8–15 Hz) was clearly modulated prior to cue presentation as well as during saccade preparation (Figs. 3 and 4c). As we previously found that many striatal neurons exhibited ramping activity before self-timed saccades^7,8^, we searched for time-variable LFP components during the self-timed period by computing Spearman’s rank correlation between time and spectral power or the magnitude of event-related potentials (ERP) for the medium and long interval conditions (for detail, see Methods). We excluded the data for the short interval condition because the time-variable changes in spectral power would be contaminated by cue-evoked response that largely spanned the self-timed period (301–444 ms). Although correlation coefficients for the power of higher frequency components (30–40 Hz) were indistinguishable from zero in all six cases (one-sample *t*-test, *t*_12*–*15_ = 0.13–1.86, *p* > 0.08, two interval conditions in three animals), the power at low frequencies (<25 Hz) consistently altered as a function of time, and correlation coefficients for the power at 8–15 Hz were statistically different from zero in all six cases (*t*_12*–*15_ = 3.10–47.7, *p* < 0.01, Fig. 4b, d, filled symbols).

**Fig. 5:**
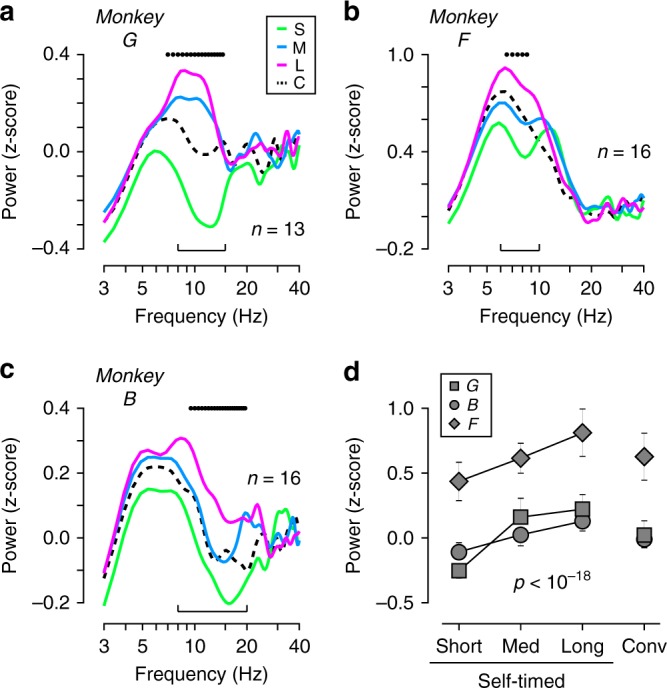
Spectral analysis of LFP during the pre-cue period. **a–c** Power spectra for monkeys G (**a**, *n* = 13 sites), F (**b**, *n* = 16), and B (**c**, *n* = 16). On each panel, black dots above the traces represent a significant difference across the interval conditions (one-way factorial ANOVA, 2 Hz bin with 0.5 Hz step, *p* < 0.01, see Methods). Brackets denote the frequency bands used for further quantification. **d** Comparison of the powers of low-frequency components. Different symbols denote different animals. Error bars indicate 95% CIs. The LFP power differed significantly across the interval conditions (repeated measures ANOVA, *F*_2,88_ = 71.5, *p* < 10^−18^)

As evident in Fig. 3 (left panels), the power of low-frequency components before the cue appearance was greater for longer interval conditions in the self-timed task. To quantify this, we computed the power spectra during 500 ms before the cue onset for each monkey (Fig. 5a–c). Despite certain individual differences, we found a striking similarity in the modulation of low-frequency components across monkeys. For all animals, the power of low-frequency components (6–20 Hz) was large for the long interval condition (magenta traces) and was small for the short interval condition (green). The difference in power between the long and short conditions showed a peak at 10.7, 13.7, and 7.8 Hz for monkeys G, B, and F, respectively. To quantify the magnitude of spectral modulation in each of the 45 sites, we computed the means of spectral power at specific frequency bands for each monkey (Fig. 5a–c, brackets) and compared them across the three interval conditions (Fig. 5d). A one-way repeated measures ANOVA revealed a significant difference (*F*_2,88_ = 71.5, *p* < 10^−18^), and post hoc paired *t*-tests indicated that the power of low-frequency components was different between any pair of interval conditions (*t*_44_ = 5.35–11.05, *p* < 10^−5^).

One might argue that these results could merely reflect volume conduction from distant brain structures, such as the thalamus and the cerebral cortex. To exclude this possibility, we also performed the same analysis on the limited sets of data obtained from offline referencing between the nearby electrodes (3–6 mm apart, Methods). In these cases, the normalized (*z*-score) power averaged 0.09 ± 0.21 (SD, *n* = 7), 0.41 ± 0.32, and 0.58 ± 0.39 for the short, medium, and long interval conditions, respectively. These values were significantly different (repeated measures ANOVA, *F*_2,12_ = 29.2, *p* < 10^−4^). Thus, the interval-dependent power modulation reflected relatively localized neural events and was unlikely to be the result of volume conduction from the overlying cerebral cortex.

We next examined the relationship between the power of low-frequency components and the magnitude of visually evoked potentials, both of which were modulated depending on the interval conditions. Correlation coefficients measured for individual recording sites averaged –0.43 ± 0.47 and were significantly different from zero (*n* = 45 sites, one-sample *t*-test, *t*_44_ = 6.08, *p* < 10^−6^). Furthermore, when we computed the normalized difference in values (power and voltage) between the short and long conditions (see Methods), we found that the effects on the evoked response and the spectral power significantly correlated (Spearman *r*_s_ = –0.31, *p* = 0.04, *n* = 45). In other words, sites with greater difference in spectral power just before the cue presentation between the conditions also exhibited greater difference in visual response gain, suggesting a possible causal link between them.

### Power modulation associated with stochastic variation in saccade latency

We previously found that pupil diameter during the pre-cue period was predictive of subsequent saccade latency in each interval condition^41^. We therefore asked whether the spectral modulation during the same pre-cue period (500 ms) also correlated with trial-by-trial saccade latencies. To test this, for each of the three interval conditions, trials with contraversive saccades were divided into two groups according to saccade latency (median split), and the spectral power at the same frequency ranges as in Fig. 4a–c (brackets) was compared. We found a significant difference in low-frequency components in the long interval condition, which averaged 0.35 ± 0.40 (SD) and 0.46 ± 0.54 for early and late contraversive saccades, respectively (*n* = 45 sites, two-tailed paired *t*-test, *t*_44_ = 2.45, *p* < 0.02). However, we failed to find any significant difference in other interval conditions (0.06 ± 0.40 versus 0.06 ± 0.38 for short and 0.31 ± 0.44 versus 0.24 ± 0.41 for medium interval conditions, respectively; *t*_44_ = 0.12 and 1.08, *p* > 0.28) or for trials with ipsiversive saccades (*t*_44_ = 0.15–0.79, *p* > 0.43, for all three interval conditions). Thus, despite clear association with the interval condition, the spectral power did not generally reflect trial-by-trial latency in each condition.

### Dissociation from the effects of reward amount on striatal LFP

Neuronal signals in the caudate nucleus are known to be modulated by the amount of reward^42^. Because of the temporal discounting of subjective value^43^, the subjective amount of reward for each correct response might be smaller for a longer interval condition. Therefore, the difference across interval conditions shown in Fig. 5 could be largely attributed to the difference in the subjective amount of reward. To address this possibility, we examined spectral power in a separate block of trials in two monkeys (B and F) where the conventional MS tasks with two different reward schedules were pseudo-randomly presented (Fig. 1b). Animals distinguished reward conditions as evident from the observation that saccade peak velocity differed between the Reward ×1 and ×2 trials (406 ± 105°/s versus 422 ± 107°/s, *n* = 24 [2 saccade directions for 12 sessions], paired *t*-test, *t*_23_ = 2.82, *p* < 0.01). In monkey B, the power at the same frequency band as in Fig. 5c (bracket) was statistically different between the reward conditions (*n* = 5, paired *t*-test, *t*_4_ = 3.63, *p* = 0.02) as well as between the long and short interval conditions (*t*_4_ = 6.97, *p* < 0.01). In monkey F, the low-frequency power was comparable between the reward conditions (*n* = 9 sites, paired *t*-test, *t*_8_ = 1.86, *p* = 0.10) but was different between the two interval conditions (*t*_8_ = 4.77, *p* < 0.01).

**Fig. 6:**
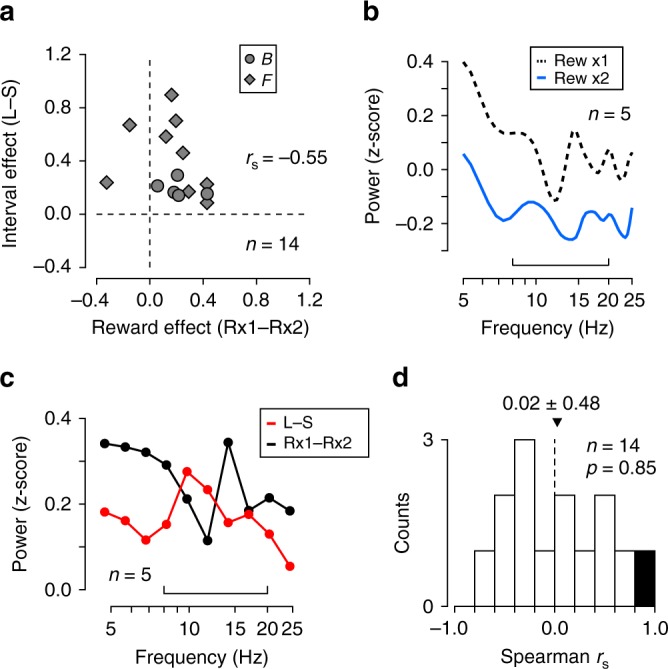
Effects of reward amount on the striatal LFP. **a** Comparison between the effects of reward amount and delay interval. Each point represents single site and compares the alteration in power at the low-frequency bands indicated by brackets in Fig. 5b, c. **b** Effects of reward amount for a broader frequency band in monkey B (*n* = 5 sites). The bracket indicates the frequency band used for the analysis in **a**. **c** Comparison of spectral modulation associated with interval condition (red, Long minus Short) with that associated with reward amount (black, Reward × 2 minus × 1). Data were divided into 10 groups. **d** Distribution of rank correlation coefficients computed for the two power spectra shown in **c** for individual sites in two monkeys. The black bar indicates statistically significant correlation (*p* < 0.05). Overall, the values were not statistically different from zero (two-tailed *t*-test, *t*_13_ = 0.19, *p* = 0.85)

Figure 6a summarizes the data from the two monkeys (*n* = 14 sites). Both the reward effects (defined as the modulation of low-frequency power, Reward ×1 minus ×2) and the interval effects (Long minus Short) were statistically different from zero (*n* = 14, one-sample *t*-test, *t*_13_ = 3.12 and 5.19, *p* < 0.01). However, these values negatively correlated (*r*_*s*_ = −0.55, *p* < 0.05), indicating that the interval effects were not merely a byproduct of the reward effects, because we would expect positive correlation in that case (i.e., sites with greater interval effects would also show greater reward effects). Thus, the interval effects were unlikely to be a simple reflection of alteration in subjective amount of reward.

To further examine this issue, we also compared the effects of two different manipulations on the power at broader frequencies. Figure 6b displays the mean spectral power for different reward conditions in the conventional MS task in monkey B (*n* = 5 sites). The effects of reward amount can be observed beyond the frequency range for the analysis in Fig. 6a (bracket, same frequency range defined in Fig. 5c). To quantify this, the difference of two power spectra was binned into 10 frequency groups (Fig. 6c, black dots) and was compared with the difference between the short and long conditions for the data obtained from the same recording sites (red). For this pair of data, the effects of two manipulations (reward and delay interval) did not correlate significantly (Spearman *r*_s_ = −0.09, *p* = 0.81). For individual recording sites in two monkeys, these effects showed a significant positive correlation only for 1 of 14 recording sites (Fig. 6d, black bar). Overall, correlation coefficients averaged 0.02 ± 0.48 and were not significantly different from zero (one-sample *t*-test, *t*_13_ = 0.19, *p* = 0.85), indicating that the reward and interval conditions differently affected the striatal LFPs. Accordingly, we conclude that the changes in spectral power across interval conditions cannot be solely explained by the possible alteration in subjective reward value resulting from temporal discounting.

Finally, we examined the time course of low-frequency power before the cue appearance. Supplementary Figure 3 shows that the abovementioned interval effects appeared 370–880 ms following the interval instruction (downward arrows), indicating that they did not merely reflect visual response to the changes in color and shape of the FP. Rather, the timing of interval effects was likely to be associated with the timing of visual cue that was presented 800−1700 ms following the instruction.

## Discussion

In this study, we examined LFPs in the antero-dorsal aspect of the caudate nucleus in monkeys performing an oculomotor version of a time production task. We found that the magnitude of visually evoked response depended on the interval condition (Fig. 2), indicating that the gain of signal transmission in the striatum had altered even before the animals started to monitor the passage of time. While the effect of interval condition was consistent across monkeys, there was considerable difference in LFP traces (Fig. 2a); this might reflect difference in relative amounts of projections from multiple cortical sources, or might simply reflect individual difference of long-latency ERP components previously reported in monkeys^44^. Along with the time-dependent modification during the self-timed period (Figs. 3 and 4), we also found that the power of low-frequency LFP components (<25 Hz) just before the cue onset displayed a clear change depending on the delay interval (Fig. 5). Because we obtained similar results even when the same analysis was performed on the selected sets of data recorded from local bipolar electrodes, these findings were not attributed to the volume conduction from distant brain structures. We also found that the pattern of alteration in spectral power for the interval condition was qualitatively and quantitatively different from those for the amounts of reward (Fig. 6), excluding the possibility that the effects of interval timing reflected the alteration of subjective reward value resulting from temporal discounting.

We found that the power at low frequencies (<25 Hz) but not at higher frequencies (30–40 Hz) showed time-dependent modification during saccade preparation (Fig. 4). This might be relevant to the recent findings that electrical stimulation to the subthalamic nucleus (STN) in PD are effective to timing behavior only when stimulation pulses are delivered at low frequency^45^. The alteration of low-frequency LFP components in the striatum has also been reported during rhythmic tapping in monkeys^46,47^ and during time production in rodents^48^. The present results extended these previous findings by showing the gradual change of spectral power during the period with no motor response. Because neurons with ramping activity were often found at or close to the present recording sites^7,8^, the spectral modulation may be relevant to such neuronal activity. However, this does not necessarily mean that the changes in power directly reflect increase or decrease in overall firing rates. Indeed, a gradual increase in power was found during self-timed period in the long interval condition in monkey F, while gradual decrease was consistently observed for the other condition and animals (Fig. 4b, d). Thus, the changes in spectral power neither represent elapsed time nor provide direct outputs for motor preparation. Instead, given that the ramping neuronal activity can be generated by integrating transient time-variable signals^16^, the low-frequency LFP components during saccade preparation may reflect online control of such integration processes within the cortico-basal ganglia pathways.

We also found clear changes in visually evoked potentials (Fig. 2) and the power of low-frequency LFP components just before the cue onset (Fig. 5). For longer delay intervals, the visual response became smaller while the LFP power at 6–20 Hz became greater. Consistent with the latter finding, a recent study has shown that human EEG demonstrates greater power at 15–30 Hz during the production of longer time intervals^49^. The cue-evoked response contained a brief ~5 Hz component (Figs. 2a and 3), which may be relevant to the previous findings that interval onset signals elicits ~4 Hz oscillations in rodent striatum^19,48^. Since low-frequency oscillation in the basal ganglia has been suggested to reflect suppression of signal transmission through the cortico-basal ganglia pathways^28,37,38^, the modulation of low-frequency components found in the present study might be causally related to the alteration of visual response. For example, during preparation for the measuring of a longer interval, the local neuronal processes relevant to the elevation of low-frequency components might suppress cortico-striatal transmission, leading to a reduced visual response. This hypothesis is consistent with the present findings that the magnitude of visual response correlated with the power of low-frequency components just before the cue presentation.

The flexible alteration of network state may be important for the adjustment of action timing. In the self-timed task, ramping up of neuronal firing rate during the self-timed period in the caudate nucleus showed different temporal profiles depending on the instructed intervals; neuronal activity exhibited more rapid accumulation (i.e., steeper slope) for shorter intervals while it reached a similar level just before movements for different intervals^7^. If low-frequency oscillations were associated with reduced cortico-basal ganglia transmission, they would attenuate visual response and also slow down the evolution of neuronal signals (i.e., lower rate of accumulation). Through this mechanism, the network state associated with low-frequency oscillations may regulate action timing by modifying the temporal profile of preparatory activity. Similar mechanism might also be at work for inhibitory control, in which both ramping activity^50^ and low-frequency oscillations^51^ have been implicated; the preceding network state with elevated low-frequency oscillations may retard the subsequent development of ramping activity, which is favorable to delay or inhibit motor response^50,52,53^. In addition to the temporal shrinkage or extension of ramping profile, the above discussion can be extended to other, more complex patterns of neuronal activity, as recent studies have shown that changes in the magnitude of input signals to a recurrent network can modulate the speed of overall evolution of neuronal activity^26,54^.

In the self-timed task, animals were required to flexibly alter the behavioral set for different intervals depending on the instructions. Previous studies have shown that in such a situation, the estimate of time interval falls under Bayesian inference and tends to be biased toward the mean of possible intervals to be presented^55,56^. This tendency seems to be exaggerated in subjects with PD (so-called migration effect)^3,57,58^. Given the fact that these subjects exhibit a sustained elevation of low-frequency components, they may have a difficulty in flexibly setting the gain of transmission in the cortico-basal ganglia loop to adjust movement timing in each trial. Indeed, while application of 10-Hz electrical stimulation to the STN in PD enhances the migration effect^58^, dopamine replacement therapy ameliorates abnormal oscillatory activity^28^ and diminishes the migration effect^3^. Because dopamine is thought to regulate cortico-striatal information flow^59^, and because focal injection of dopamine antagonists into the striatum alters self-timing^8^, the dopaminergic signals might play a crucial role in modulating the low-frequency oscillatory activity described here.

Although the spectral power before the cue onset consistently differed across interval conditions (Fig. 5), it did not differ between trials with longer and shorter latencies in a given condition, except for one condition (contralateral saccades with long delay interval). These results indicate that the low-frequency oscillatory activity in the striatum correlated with the intended rather than the actual interval timing to be reported. In sharp contrast, we have previously shown that pupil diameter before the cue onset inversely correlates with trial-by-trial latencies in each of different interval conditions but not with saccade latencies across different conditions^41^. These results suggest that the brain may implement at least two distinct timing mechanisms; one intentionally determines the action timing depending on the context, while the other is responsible for the implicit, stochastic variation of timing^7,60^. Previous studies have shown that pupil size strongly correlates with neuronal activity in the locus coeruleus (LC)^61^. The LC sends noradrenergic projections to widespread brain regions^61^, whereas those to the basal ganglia are exceptionally sparse^62^. These previous observations suggest that the basal ganglia may not be within the timing system that is linked with pupil diameter and stochastic variation, whereas the cortico-cerebellar system might be involved. The cerebellum receives massive noradrenergic projections^63^, and neuronal firing during the self-timed period in the cerebellum closely tracks trial-by-trial latencies of self-timed saccades in the range of hundreds of milliseconds^64^. The idea of two different timing mechanisms is also supported by a recent study in rodents showing that different areas in the frontal cortex introduce overall bias of action timing and stochastic trial-by-trial variation, respectively^60^.

Our results were also consistent with the recent findings of categorical timing signals. Previous studies have demonstrated that neuronal representation of timing in the dorsomedial frontal cortex^14,65^ and the inferior parietal cortex^66^ is tuned for specific intervals. Furthermore, the tuned representation in neurons in the putamen during rhythmic tapping has been shown to be biased toward longer intervals in subsecond range^46^. The LFP power modulation found in the present study might be relevant to such categorical representation of interval timing, while stochastic variation may originate from neuronal signals outside of the cortico-basal ganglia networks.

In summary, we have demonstrated that low-frequency LFP components in the primate caudate nucleus were modulated during the time production task. Alteration of the network state within the cortico-basal ganglia system may be important for temporal control of actions. Future studies with a more direct assessment of specific neuronal circuits will elucidate the underlying mechanism in detail.

## Methods

### Animal preparation and surgery

Subjects were three adult Japanese monkeys (*Macaca fuscata*, one male and two females, aged 6–14 years, 6–9 kg). All experimental protocols were evaluated and approved in advance by the Hokkaido University Animal Care and Use Committee. In separate surgical procedures, animals were sterilely implanted with head holders, eye coils, and recording chambers under general isoflurane anesthesia. Analgesics (pentazocine and ketoprofen) were administered during and a few days following each surgery. After full recovery, the animals were trained on the oculomotor tasks. During the training and experimental sessions, animals sat in a primate chair in a dark booth with their heads restrained. Horizontal and vertical eye position were recorded using the search coil technique (MEL-25, Enzanshi Kogyo).

### Visual stimuli and behavioral tasks

Experiments were controlled using a real-time data acquisition system (TEMPO, Reflective Computing). Visual stimuli were presented on a 24-inch cathode ray tube monitor (refresh rate: 60 Hz) or a 27-inch liquid crystal display monitor (refresh rate: 144 Hz) located 38 or 46 cm from the eyes (64 × 44° and 66 × 40° of visual angle, respectively). In the self-timed saccade task (Fig. 1a), each trial began with the appearance of an FP (white 0.5–0.7° square) at the center of the screen. When eye position remained within 3° of the FP for 400 ms, the color (and shape) of the FP changed to instruct the interval condition. Specifically, a cyan unfilled square, a blue filled square, and a yellow filled square indicated short (400 ms), medium (1000 ms), and long (2200 ms) mandatory delay intervals, respectively, for monkeys B and G. For monkey F, a pink square, a green triangle, and a cyan unfilled square were assigned to short, medium, and long intervals, respectively. After a variable delay (800–1700 ms), a visual cue (white 0.5–0.7° square) was presented 16° right or left of the FP for 100 ms. Animals received a juice reward when they made a saccade to the cue location after the instructed interval and maintained fixation for ≥800 ms on the target that reappeared immediately after the saccade. Trials were aborted if monkeys were unable to maintain fixation until the cue onset, or failed to generate self-initiated saccades until 800, 1700, or 3200 ms following the cue onset (only 1.2% trials). Saccade timing was detected online as eye position deviated >3–4° from the FP. In the conventional MS task (Fig. 1b), the FP disappeared after a variable delay (1000–2400 or –2500 ms for monkey G, 800–2400 ms for monkeys B and F) following the cue onset. Animals were required to generate a saccade to the cue location within 600 ms to obtain a liquid reward. The timing of reward delivery was identical to the self-timed saccade task, except for four sessions in which the reward was delivered 1400 ms after the FP offset.

Data were obtained from 41 experimental sessions (*n* = 13, 14, and 14 sessions in monkeys G, B, and F, respectively), in which three delay interval conditions of the self-timed task and the conventional MS task were presented pseudo-randomly (four different conditions in two directions). In most experiments, the same amount of reward was delivered for all conditions. However, in a subset of experiments (5 and 7 sessions in monkeys B and F), we also presented a separate block consisted of only conventional MS tasks with different reward schedule (Reward ×1 and ×2). In the Reward ×2 trials, the reward amount was approximately doubled. The reward amounts were associated with different FP colors; Reward ×1 and Reward ×2 conditions were associated with red and purple squares (monkey B) or yellow and red circles (monkey F), respectively.

### Physiological procedures

LFPs were recorded from the sites in the caudate nucleus where we previously found neurons with preparatory activity for self-timed saccades (Fig. 2b and Supplementary Figure 1)^7,8^. According to the previous anatomical studies, our recording sites seemed to be close to the regions receiving inputs from both the dorsolateral and dorsomedial frontal cortex^67–69^. In most experiments (38 of 41 sessions), a single tungsten electrode (FHC or Alpha Omega) was inserted into the right (monkey B) or left (monkeys G and F) caudate nucleus through a 23-gauge stainless-steel guide tube using a micromanipulator (MO-97S; Narishige). In one session in monkey F, we simultaneously recorded the activity of three single electrodes (3–4 mm apart from each other). In two sessions in monkey B, a linear array electrode made from four nichrome wires (2-mm spacing) were inserted so that two contacts were located within the caudate nucleus and the others were at the border (not used for analysis) and 2 mm above the nucleus (used for offline re-referencing; see the next section). Signals were referenced to either the guide tube or Ag-AgCl disc electrode placed on the dura mater and were amplified and filtered (1–100 or 1–300 Hz). During seven experiments, we also used a notch filter centered at 50 Hz to remove line noise. Data were obtained from a total of 45 recording sites (*n* = 13, 16, and 16 sites, for monkeys G, B, and F, respectively) in 41 experimental sessions.

### Experimental design and statistical analysis

Data of eye position and neural signals were digitized at 16-bit resolution and sampled at 1 kHz, and were saved in files during the experiments along with event timestamps. Data were analyzed offline using MATLAB (MathWorks). Trials with goal non-directed saccades (>8° from the cue location, 8.4%) were excluded from the analysis. For the self-timed saccade task, trials were removed when the saccade latency was shorter than 60% of the mandatory delay intervals (6.3%). For the conventional MS task, the error trials (mostly due to a fixation break before the FP offset) and early anticipatory saccades (reaction time <70 ms) were discarded. The LFPs were assessed during the period starting from 200 ms before fixation to 100 ms after saccade initiation, and the trials with unusual noise (exceeding or below the median ±140 μV) were removed from further analysis (2.3%).

For the analysis of the ERP, the LFPs were aligned on the cue onset and averaged over trials after subtracting the mean value measured during 500 ms prior to the cue onset (Fig. 2a). For each condition in each site, we measured the magnitude of the visually evoked response as the mean during an 11-ms period centered at the maximal response, which was assessed during the 100–200 ms (monkey G) or 150–300 ms (monkeys B and F) period from the cue onset. For comparison across sessions, the data were normalized for each site so that the maximal and minimal values became 1 and 0, respectively (Fig. 2b). To do this, we used the formula, (*x*−min)/(max−min), where *x* represents the response in a given condition and max and min indicate the maximal and minimal values, respectively.

Discrete Fourier transform was used to obtain spectrograms in Fig. 3 and Supplementary Figure 2. We applied fast Fourier transform (FFT) to the LFP data using a 300-ms sliding window with 10-ms steps, tapered by a Hamming window. The estimated power at each frequency was *z*-scored according to the mean and SD of the power measured during the 400-ms pre-instruction period (i.e., before the FP color change) for each recording site and then was averaged across multiple sites. This method was used for display purposes only. Although the previous studies reported ~4 Hz oscillation in the basal ganglia during the timing task in rodents^19,48^ and humans^45^, our window size was too short to evaluate such slow fluctuations; instead, we were able to assess LFP modulation during relatively short, discrete task events. Nevertheless, we also found similar oscillations just after the cue onset (Figs. 2a and 3, see Discussion). The time courses of the LFP power shown in Fig. 4 and Supplementary Figure 3 were computed by Hilbert transform following Butterworth filtering (4th order) in both directions (forward and backward in time). As before, the data were *z*-scored according to the values during the pre-instruction period. Spearman’s rank correlation coefficients between time and power (Fig. 4b, d) were calculated for the period starting at the mean of the cue onset time and ending at 100 ms before saccades (i.e., data during the self-timed period). For this analysis, the data before the cue onset were removed, and then the data were grouped into 10 equally spaced temporal bins. To obtain the spectral power during the pre-cue period (500 ms; Fig. 5), we applied FFT to the LFPs with a Hamming window. For each site and each block, the power at each frequency was *z*-scored according to the data during the 500-ms baseline period starting from 400 ms before the instructions. Following the previous studies^46,47^, we also constructed Figs. 3 and 5 using a Slepian taper (Chronux toolbox, ver. 2.10, http://chronux.org)^70^ with the bandwidth parameter of 10 Hz, yielding time-bandwidth products of 3 and 5 for 300- and 500-ms windows, respectively. The resulting spectrograms and power spectra were very similar to the original ones in Figs. 3 and 5, respectively, and the statistical results in Figs. 5d and 6 remained essentially unchanged except that the number of sites with significant correlation in Fig. 6d (black bar) became four (one negative and three positive values).

For the spectral analysis of the data from simultaneous recordings from multiple sites, the data were re-referenced offline to minimize the effects of volume conduction. In these cases, the signal from an electrode in the caudate nucleus was subtracted by that obtained from the reference electrode in the white matter (monkey B, 2 mm above the border of the caudate nucleus) or the averaged signal of the other two electrodes (monkey F). Unless otherwise stated, these data (*n* = 4 and 3 sites for monkeys B and F, respectively) were combined with the remaining data from 38 sites with a single electrode because this procedure did not alter the main results.

For the analysis of the relationship between the magnitude of visually evoked response (μV) and the low-frequency power modulation before the cue onset (*z*-score), we calculated Pearson’s correlation coefficient across four conditions in each recording site. We also computed the normalized (*z*-scored) differences between the short and long interval conditions for each monkey and then assessed Spearman’s correlation coefficients across 45 sites.

For the statistical analyses, we performed one-way repeated measures ANOVAs followed by post hoc two-tailed paired *t*-tests for comparisons across interval conditions, and unpaired or paired *t*-tests for group comparisons. In Fig. 5a–c, we compared the data across three interval conditions using one-way factorial ANOVAs (2 Hz window with 0.5 Hz step) to indicate frequencies with a statistical difference. To obtain the corrected critical *p*-value at an alpha level of 0.05, we conducted permutation tests. For each monkey, self-timed trials were shuffled without changing the number of trials for each condition, and the minimal *p*-value across all bins (75 frequency bands) was computed. We performed 1000 iterations, and then obtained the fifth percentile values that were 0.0119, 0.0362, and 0.0185 for monkeys G, B, and F, respectively. According to these results, we took 0.01 as the critical *p*-value to indicate the statistically significant interval effects shown in Fig. 5a–c (black dots).

### Reporting Summary

Further information on experimental design is available in the Nature Research Reporting Summary linked to this article.

## Supplementary information

Supplementary Information

Description of Additional Supplementary Files

Reporting Summary

Supplementary Data 1

## Data Availability

The data that support the findings of this study are available from the authors upon reasonable request. All source data underlying the graphs presented in the main figures is available as Supplementary Data 1.
