# Supplementary material for: Neural oscillations in the primate caudate nucleus correlate with different preparatory states for temporal production
Source: Commun Biol. 2019 Mar 14;2:102. doi: 10.1038/s42003-019-0345-2 (PMC6418172; doi:10.1038/s42003-019-0345-2)
Supplement: Supplementary file 1 — Supplementary Information [file 42003_2019_345_MOESM1_ESM.pdf]

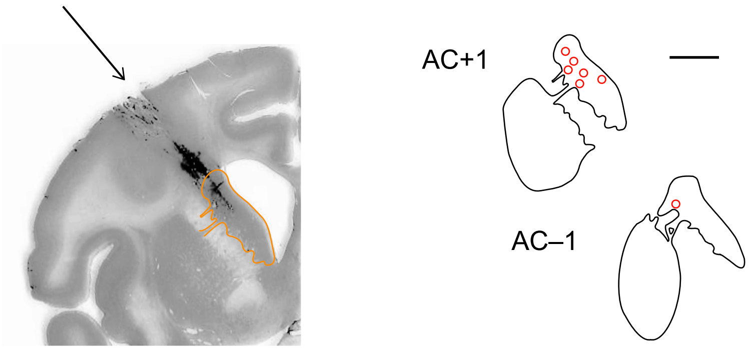

Supplementary Figure 1. Histological section and recording sites in monkey G. Scale bar indicates 5 mm. Coronal sections at the level of 1 mm anterior and posterior to the anterior commissure (AC) are shown.

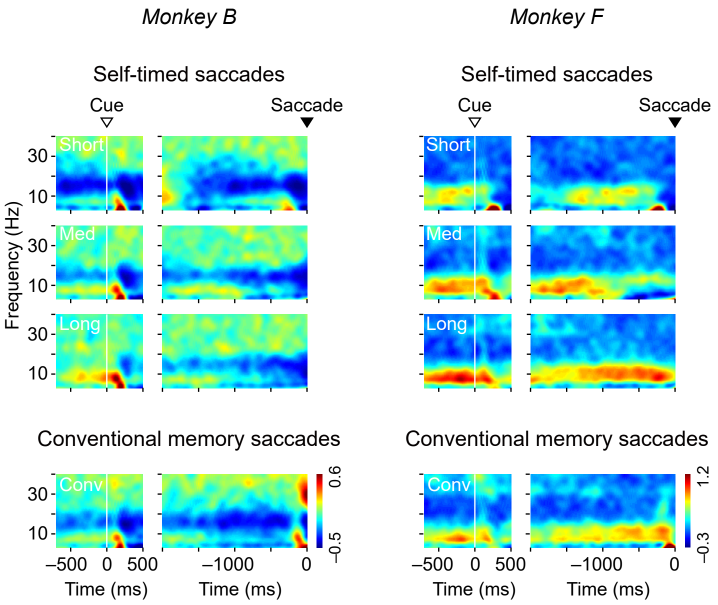

Supplementary Figure 2. Color-coded power spectra of LFPs for monkeys B and F (n = 16 sites for each). Convention of figure is same as Figure 3.

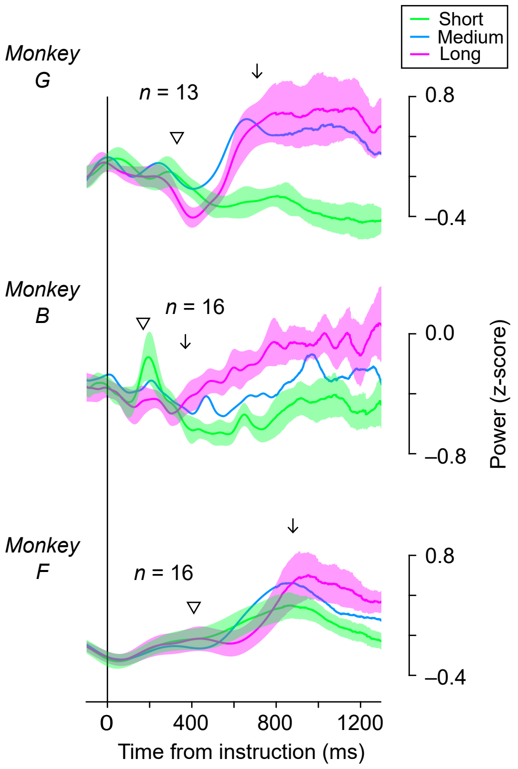

Supplementary Figure 3. Time courses of the power of low-frequency components aligned with the instruction onset during fixation ($n = 13$ or 16 sites). Data following the cue onset were removed before averaging data across trials. Timing of power modulation across three interval conditions were assessed by repeated measures ANOVA for every 20 ms (10 ms step), and the times when more than five consecutive bins started to be statistically different ($p < 0.01$) are indicated by inverted triangles. Downward arrows indicate the times when the power becomes consistently ordered (short < medium < long). Note that the visual cue appeared 800−1700 ms following the instruction. Shaded areas indicate 95% CIs and are shown for the short and long interval conditions only.
