# Supplementary material for: Neural oscillations in the primate caudate nucleus correlate with different preparatory states for temporal production
Source: Commun Biol. 2019 Mar 14;2:102. doi: 10.1038/s42003-019-0345-2 (PMC6418172; doi:10.1038/s42003-019-0345-2)
Supplement: Supplementary file 2 — Description of Additional Supplementary Files [file 42003_2019_345_MOESM2_ESM.docx]

**Description of Additional Supplementary Files**

**File Name**: Supplementary Data 1

**Description**: Numerical data for Figures 1, 2, 4, 5 and 6
