# Supplementary material for: Neural oscillations in the primate caudate nucleus correlate with different preparatory states for temporal production
Source: Commun Biol. 2019 Mar 14;2:102. doi: 10.1038/s42003-019-0345-2 (PMC6418172; doi:10.1038/s42003-019-0345-2)
Supplement: Supplementary file 3 — Reporting Summary [file 42003_2019_345_MOESM3_ESM.pdf]

nature research

Corresponding author(s): Masaki Tanaka

Last updated by author(s): Dec 17, 2018

Reporting Summary

Nature Research wishes to improve the reproducibility of the work that we publish. This form provides structure for consistency and transparency in reporting. For further information on Nature Research policies, see Authors & Referees and the Editorial Policy Checklist.

Statistics

For all statistical analyses, confirm that the following items are present in the figure legend, table legend, main text, or Methods section.

n/a	Confirmed	
☐	☒	The exact sample size (n) for each experimental group/condition, given as a discrete number and unit of measurement
☐	☒	A statement on whether measurements were taken from distinct samples or whether the same sample was measured repeatedly
☐	☒	The statistical test(s) used AND whether they are one- or two-sided<br>*Only common tests should be described solely by name; describe more complex techniques in the Methods section.*
☐	☒	A description of all covariates tested
☐	☒	A description of any assumptions or corrections, such as tests of normality and adjustment for multiple comparisons
☐	☒	A full description of the statistical parameters including central tendency (e.g. means) or other basic estimates (e.g. regression coefficient) AND variation (e.g. standard deviation) or associated estimates of uncertainty (e.g. confidence intervals)
☐	☒	For null hypothesis testing, the test statistic (e.g. F, t, r) with confidence intervals, effect sizes, degrees of freedom and P value noted<br>*Give P values as exact values whenever suitable.*
☒	☐	For Bayesian analysis, information on the choice of priors and Markov chain Monte Carlo settings
☒	☐	For hierarchical and complex designs, identification of the appropriate level for tests and full reporting of outcomes
☒	☐	Estimates of effect sizes (e.g. Cohen's d, Pearson's r), indicating how they were calculated

Our web collection on statistics for biologists contains articles on many of the points above.

Software and code

Policy information about availability of computer code

Data collection	TEMPO system (Reflective Computing)
Data analysis	MATLAB (Mathworks)

For manuscripts utilizing custom algorithms or software that are central to the research but not yet described in published literature, software must be made available to editors/reviewers. We strongly encourage code deposition in a community repository (e.g. GitHub). See the Nature Research guidelines for submitting code & software for further information.

Data

Policy information about availability of data

All manuscripts must include a data availability statement. This statement should provide the following information, where applicable:

- Accession codes, unique identifiers, or web links for publicly available datasets
- A list of figures that have associated raw data
- A description of any restrictions on data availability

The data that support the findings of this study are available from the authors upon reasonable request.

Field-specific reporting

Please select the one below that is the best fit for your research. If you are not sure, read the appropriate sections before making your selection.

☒ Life sciences ☐ Behavioural & social sciences ☐ Ecological, evolutionary & environmental sciences

For a reference copy of the document with all sections, see nature.com/documents/nr-reporting-summary-flat.pdf

Life sciences study design

All studies must disclose on these points even when the disclosure is negative.

Sample size	Local field potential data obtained from 45 recording sites in the caudate nuclei of three macaque monkeys.
Data exclusions	No data exclusions. Details of data collection and re-referencing of LFP data obtained from multichannel electrodes are provided in the Methods.
Replication	All findings were statistically evaluated.
Randomization	This is not applicable because this study does not contain clinical data.
Blinding	For the reason above, this is not applicable.

Reporting for specific materials, systems and methods

We require information from authors about some types of materials, experimental systems and methods used in many studies. Here, indicate whether each material, system or method listed is relevant to your study. If you are not sure if a list item applies to your research, read the appropriate section before selecting a response.

Materials & experimental systems

n/a	Involved in the study	
☒	☐	Antibodies
☒	☐	Eukaryotic cell lines
☒	☐	Palaeontology
☐	☒	Animals and other organisms
☒	☐	Human research participants
☒	☐	Clinical data

Methods

n/a	Involved in the study	
☒	☐	ChIP-seq
☒	☐	Flow cytometry
☐	☒	MRI-based neuroimaging

Animals and other organisms

Policy information about studies involving animals; ARRIVE guidelines recommended for reporting animal research

Laboratory animals	Macaque fuscata (Japanese monkeys)
Wild animals	No wild animal was used. All monkeys were raised for experiments and were provided by the National Bio-resource Project in Japan.
Field-collected samples	N.A.
Ethics oversight	The Hokkaido University Animal Care and Use Committee evaluated and approved the experimental protocols.

Note that full information on the approval of the study protocol must also be provided in the manuscript.

Magnetic resonance imaging

Experimental design

Design type	T2-weighted structural MRI for locating the recording sites.
Design specifications	Taken from anesthetized animals.
Behavioral performance measures	Not applicable. We did not obtain functional MRI.

Acquisition

Imaging type(s)	structual
Field strength	3.0 T
Sequence & imaging parameters	3D T2-weighted images with 0.5-mm slices.
Area of acquisition	*State whether a whole brain scan was used OR define the area of acquisition, describing how the region was determined.*

Diffusion MRI ☐ Used ☒ Not used

Preprocessing

Preprocessing software	N.A.
Normalization	N.A.
Normalization template	N.A.
Noise and artifact removal	N.A.
Volume censoring	N.A.

Statistical modeling & inference

Model type and settings	N.A.
Effect(s) tested	N.A.

Specify type of analysis: ☐ Whole brain ☒ ROI-based ☐ Both

Anatomical location(s): Coronal sections around the anterior commissure. The details are provided in Supplementary Figure 1.

Statistic type for inference (See Eklund et al. 2016)	N.A.
Correction	N.A.

Models & analysis

n/a	Involved in the study
☒	☐ Functional and/or effective connectivity
☒	☐ Graph analysis
☒	☐ Multivariate modeling or predictive analysis
